# Molecular Mechanism and Treatment of Viral Hepatitis-Related Liver Fibrosis

**DOI:** 10.3390/ijms150610578

**Published:** 2014-06-12

**Authors:** Tung-Hung Su, Jia-Horng Kao, Chun-Jen Liu

**Affiliations:** 1 Graduate Institute of Clinical Medicine, National Taiwan University College of Medicine, 1 Chang-Te Street, Taipei 10002, Taiwan; E-Mail: tunghungsu@gmail.com; 2 Department of Internal Medicine, National Taiwan University Hospital, 7 Chung-Shan South Road, Taipei 10002, Taiwan; 3 Hepatitis Research Center, National Taiwan University Hospital, 1 Chang-Te Street, Taipei 10002, Taiwan; 4 Department of Medical Research, National Taiwan University Hospital, 7 Chung-Shan South Road, Taipei 10002, Taiwan; 5 Taiwan Liver Disease Consortium (TLC), 7 Chung-Shan South Road, Taipei 10002, Taiwan

**Keywords:** hepatitis B, hepatitis C, hepatocellular carcinoma, myofibroblast, hepatic stellate cells, antiviral therapy, nucleos(t)ide analogs, interferon, molecular target agent, macrophage

## Abstract

Hepatic fibrosis is a wound-healing response to various chronic stimuli, including viral hepatitis B or C infection. Activated myofibroblasts, predominantly derived from the hepatic stellate cells (HSCs), regulate the balance between matrix metalloproteinases and their tissue inhibitors to maintain extracellular matrix homeostasis. Transforming growth factor-β and platelet-derived growth factor are classic profibrogenic signals that activate HSC proliferation. In addition, proinflammatory cytokines and chemokines coordinate macrophages, T cells, NK/NKT cells, and liver sinusoidal endothelial cells in complex fibrogenic and regression processes. In addition, fibrogenesis involves angiogenesis, metabolic reprogramming, autophagy, microRNA, and epigenetic regulations. Hepatic inflammation is the driving force behind liver fibrosis; however, host single nucleotide polymorphisms and viral factors, including the genotype, viral load, viral mutation, and viral proteins, have been associated with fibrosis progression. Eliminating the underlying etiology is the most crucial antifibrotic therapy. Growing evidence has indicated that persistent viral suppression with antiviral therapy can result in fibrosis regression, reduced liver disease progression, decreased hepatocellular carcinoma, and improved chances of survival. Preclinical studies and clinical trials are currently examining several investigational agents that target key fibrogenic pathways; the results are promising and shed light on this debilitating illness.

## 1. Introduction

Fibrotic diseases account for up to 45% of the total deaths in developed countries [1]. However, active antifibrotic therapies for established cirrhosis are currently unavailable, leaving the needs of cirrhosis patients unmet. Understanding the molecular mechanisms of liver fibrogenesis can shed light on how to ameliorate this debilitating disease.

Chronic hepatitis B and C are major causes of liver fibrosis progression and cirrhosis worldwide. Long-term hepatic necroinflammation is the main contributor to both fibrogenesis and carcinogenesis of the liver. However, growing lines of evidence indicate that successful antiviral therapy may halt or reverse liver fibrosis, especially in the early stages. Several molecular target agents (e.g., sorafenib) have been shown to improve experimental liver fibrosis. Targeted therapies using these agents against fibrogenesis may be the next step in the discovery of an antifibrotic treatment.

In this paper, we review and discuss the pathobiology of liver fibrosis, the clinical aspects and molecular mechanisms of viral-hepatitis-related fibrosis and cirrhosis, and the benefits of antiviral therapy. Finally, we evaluate and explore potential molecular target therapies for liver fibrosis.

## 2. Pathobiology for Liver Fibrogenesis and Regression

Hepatic fibrosis is a wound-healing response to various chronic stimuli, including hepatitis viral infection, metabolic disorders, alcohol abuse, and autoimmune attacks in liver (Table 1). During the course of fibrogenesis, various mediators, which are mainly produced by Kupffer cells, resident hepatic cells, and infiltrating inflammatory cells,, activate myofibroblasts, causing excess extracellular matrix (ECM) accumulation. Fibrosis is the resulting imbalance between ECM production and resolution. The excessive ECM deposition (especially type 1 collagen deposition) disorders the normal architecture of the liver, resulting in fibrosis progression and subsequent cirrhosis [2]. At least five responses to injury-induced functional or physical disruption of epithelial cells can provoke tissue fibrosis, including cell death, dysregulation of metabolic pathways (resulting in cell stress, endoplasmic reticulum stress, and the generation of reactive oxygen species), epithelial-to-mesenchymal transition (EMT), transforming growth factor (TGF)-β activation, and immunological responses [3].

**Table 1:** Causes of fibrosis and specific therapies for underlying etiology.

Causes of Fibrosis	Specific Therapies for Underlying Etiology
Hepatitis B	Conventional and pegylated interferon, lamivudine, adefovir, telbivudine, entecavir, tenofovir
Hepatitis C	Conventional and pegylated interferon/ribavirin, boceprevir, telaprevir, simeprevir, sofosbuvir, daclatasvir/asunaprevir
Steatosis	Lifestyle modification, obeticholic acid
Alcohol	Alcohol abstinence
Autoimmune	Prednisolone, azathioprine
Primary biliary cirrhosis	Ursodeoxycholic acid
Primary sclerosing cholangitis	Ursodeoxycholic acid
Hemochromatosis	Therapeutic phlebotomy, deferoxamine
Wilson’s disease	Penicillamine, trientine hydrochloride, zinc
Schistosomiasis	Praziquantel

### 2.1. The Major Factor of Fibrogenesis: Myofibroblasts and Hepatic Stellate Cells

The key factor of fibrogenesis, myofibroblasts, are highly proliferative cells that exhibit enhanced survival and migrate to and accumulate at sites of liver injury in response to the autocrine and paracrine stimuli of various growth factors, cytokines, lipid mediators, or adipokines produced by the injured liver [4]. Heterogeneity among cell populations, including hepatic stellate cells, portal fibroblasts, and bone-marrow derived fibroblasts, is a relevant contributor to the myofibroblast pool [5]. Previous studies have suggested that the EMT from cholangiocytes and hepatocytes contributes to hepatic fibrosis [6]; however, novel sophisticated fate tracing experiments have discounted the contribution of these epithelial cells to myofibroblasts [7,8]. Mederacke *et al.* used a novel LratCre-transgenic mouse that marked 99% of hepatic stellate cells (HSCs), revealing that HSCs account for 82% to 96% of the myofibroblasts in models of toxic, cholestatic, and fatty liver disease [9]. Their study confirmed that HSCs are the major contributors to fibrogenesis.

HSCs account for 5% to 8% of total liver cells [10], and their functions include vitamin A homeostasis [11]; ECM synthesis and degradation; sinusoidal blood flow regulation [12]; erythropoietin expression in the perinatal period [13]; contribution to the plasminogen activation system [14]; and secretion of paracrine, juxtacrine, autocrine, and chemoattractant mediators.

Responding to various stimuli from parenchymal injury, the inflammatory reaction generates large panels of profibrogenic signals (transcriptional factors and morphogens), and, subsequently, quiescent HSCs are primed and activated by the signals of persistent tissue injury [15]. The myofibroblastic phenotype of these activated HSCs is characterized by the expression of α-smooth muscle actin (α-SMA); a parallel loss of retinoids and lipid droplets; a reduction in the expression of adipogenic/lipogenic factors; and a *de novo* expression of receptors for fibrogenic, chemotactic, and mitogenic factors [4]. The balance between matrix metalloproteinases (MMPs, ECM degrading enzymes) and tissue inhibitors of the metalloproteinase family (TIMPs) is strongly regulated by HSCs. At the early stages of fibrogenesis, HSCs express MMPs, but not TIMPs, causing the liver ECM to degrade. However, fully activated HSCs express TIMPs and inhibit MMPs, thereby inhibiting ECM degradation [16]. In addition, the ECM molecules, matrix stiffness, and collagen cross-linking promote the HSC activation process through integrin-mediated pathways [16].

### 2.2. Inflammation: The First Step of Fibrogenesis

Interaction with macrophages and inflammatory signals drives HSC activation. Lipopolysaccharide (LPS) originating from intestinal microflora can activate HSCs through the toll-like receptor 4 signaling pathway, [17], which increasingly express proinflammatory cytokines and chemokines (e.g., CCL2, CCL4, and CX3CL1) [17,18]. CCL2 (MCP-1) recruits inflammatory Gr1^+^/Ly6C^+^-expressing monocytes from the peripheral blood into the injured liver and promotes hepatic fibrosis [19], and CX3CL1 (fractalkine) protects against hepatic fibrosis by controlling the differentiation of infiltrating monocytes into proinflammatory macrophages and the survival of infiltrating monocytes [20]. CCR1 and CCR5 play distinct roles in promoting hepatic fibrosis in Kupffer cells and HSCs [21]. RANTES (regulated on activation normal T cell expressed and secreted), CCR1, and CCR5 are appreciably up-regulated in patients with hepatic cirrhosis, indicating the activation of the CC chemokine system in human fibrogenesis [21].

HSCs reside within the perisinusoidal space of Disse in close proximity to liver sinusoidal endothelial cells (LSECs), Kupffer cells, and dendritic cells; therefore, HSCs may indirectly influence the antigen-presenting function. Prior studies have demonstrated that HSCs express MHC-class II molecules and may present antigens to induce T-cell responses [22]. However, in a recent study, highly purified HSCs did not present antigens to naive MHC-II-restricted CD4 T cells [23]. HSCs function indirectly by mediating retinoid acid and TGF-β dependent regulatory T (Treg)-cell induction and the inhibition of Th17 cells primed by other antigen-presenting cells. These findings suggest that HSCs serve as regulatory bystanders that can enhance the differentiation and accumulation of regulatory T cells [23].

### 2.3. Molecular Mechanisms of Fibrogenesis

TGF-β1 is a common major profibrogenic cytokine in liver disease, promoting HSC activation, hepatocyte apoptosis, and ECM formation and inducing several profibrogenic mediators such as TIMP-1 [24]. TGF-β1 is regulated by stimulatory activators (Smad 2 and 3) and inhibitory signals (Smad 7) [24]. However, studies have suggested that the TGF-β1 secreted from Treg cells functions as an antiinflammatory and antifibrotic mediator [25]. Several clinical studies have reported that patients with chronic HBV or HCV infections have elevated TGF-β1 serum levels [26,27].

The *N*-terminal latency-associated peptide domain of TGF-β cross-links with latent TGF-β binding proteins and the ECM. Upon liver injury, cholangiocytes express high levels of integrin α_v_β_6_, which binds to the LAP portion of TGF-β1 and TGF-β3 [3]. Myofibroblast activation involves the activation of the platelet-derived growth factor (PDGF) and connective tissue growth factor (CTGF), as well as TGF-β [2].

The PDGF receptor pathway is another key mitogen in HSC proliferation [28]. PDGF stimulation can activate mitogen-activated protein kinase (MAPK) signaling involving the activation of Ras–Rac–Raf followed by the activation of mitogen-induced extracellular kinase, and extracellular signal-regulated kinase (ERK) and C-Jun nuclear kinase. In addition, PDGF stimulation can activate either the matrix-associated focal adhesion kinase or the phosphatidylinositol 3-kinase–Akt–p70S6 kinase-signaling pathway, leading to the activation of downstream kinases such as Akt and p70S6K [29].

### 2.4. Microenvironment Involving Fibrogenesis

#### 2.4.1. Macrophage Polarization

Resident and recruited macrophages, which are key components in the liver homeostasis process, have dual roles in matrix deposition and remodeling, and regulate activated myofibroblasts (and their precursor populations) and endothelial cells [3]. The activated macrophages produce TNF-α and IL-1, which, in turn, activate fibroblasts and induce the overproduction of ECM proteins [30].

Previous studies have demonstrated that the phagocytosis of apoptotic hepatocytes and cholangiocytes by macrophages can eliminate fibrosis [31,32]. Macrophages can also directly clear excess collagen [33]. In response to environmental stimulation, macrophages undergo polarized activation, attaining an M1, M2, or M2-like activation status [34]. The M1 subtype consists of a classically activated macrophages that exhibit inflammatory and microbicidal activity; the M2 subtype consists of alternatively activated macrophages, or wound-healing macrophages, that function in tissue repair and exhibit profibrotic activities; and the M2-like subtype consists of regulatory macrophages that contribute to the resolution of inflammation and fibrosis [3,35].

The key regulator of macrophage polarization belongs to the interferon regulatory factor (IRF)-signal transducer and activator of transcription (STAT)-suppressor of cytokine signaling (IRF-STAT-SOCS) families. Interferon-γ and the TLR-activated IRF-STAT signaling pathway activate the M1 phenotype through STAT1. IL-4 and IL-13 mediate STAT6 activation, and IL-3 mediates STAT5 activation to promote M2 polarization. IL-10 activates the M2-like phenotype through STAT3 [34,36]. Because of the secretion of IL-13 signals, Th2 cells can drive M2 polarization to produce TGF-β, which is activated by MMP-9 [37] and subsequently activates HSCs to promote fibrosis. The Th1 responses drive M1 polarization and are associated with antifibrotic activity [5,30]. Subpopulations of macrophages can either mediate the destruction of ECMs directly or indirectly by inducing the release of MMPs (M1) or support cellular proliferation, stimulate the production of deposited ECM proteins, and promote the synthesis and secretion of new ECM molecules (M2) [30].

Hepatic macrophages enhance myofibroblast survival in a manner dependent on nuclear factor (NF)-κB, thereby promoting liver fibrosis [38]. The proinflammatory and profibrogenic Gr1^hi^/Ly6C^hi^ monocytes express CCR1, CCR2, CCR6, CCR8, and CCR9. After activation of these receptors, the infiltrating monocytes differentiate into proinflammatory macrophages and drive HSC activation [39,40]. The Gr1^l^ monocyte subset expresses antiinflammatory and antifibrogenic chemokines, such as CXCR1 and CX3CR1 [41]. The CX3CR1-CX3CL1 interaction promotes macrophage survival, inhibits inflammatory properties in macrophages, and results in a reduction of liver inflammation and fibrosis [41]. The MMP-13 and MMP-9 produced by macrophages and dendritic cells facilitate fibrosis resolution [42,43].

#### 2.4.2. T Cells and NK/NKT Cells

CD4^+^ lymphocytes, including Th1, Th2, Th17, and Treg cells, play vital roles in immune responses controlling fibrogenesis. Th1 cells release IFN-γ, reducing liver fibrogenesis by inhibiting the TGF-β-induced transduction cascade [4]. Th2 cells promote liver fibrosis by producing IL13 [44]. The number of Th17 cells, or IL17-positive lymphocytes, increases in patients with chronic hepatitis B or alcoholic liver disease and correlates with the severity of liver fibrosis [45,46]. In one study, hepatic IL-17 levels were elevated in experimental liver fibrosis [47]. IL-17 directly induced the production of type I collagen in HSCs by activating the STAT3 signaling pathway. Furthermore, mice devoid of STAT3 signaling in HSCs (GFAP-STAT3^−/−^ mice) were less susceptible to fibrosis compared with wildtype controls [47].

In addition to antiviral and antitumor properties, liver-specific NK and NKT cells exhibit antifibrotic effects. For example, NK cells can induce apoptosis in early-activated HSCs or promote senescence through a TRAIL-medicated pathway [48].

#### 2.4.3. Liver Sinusoidal Endothelial Cells

The early activation of LSECs with loss of fenestration, production of TGF-β or PDGF-BB, and secretion of ECM proteins (e.g., type 1 collagen) contributes to HSC activation [4]. Moreover, restoration of LSEC differentiation promotes the HSC quiescence and subsequent fibrosis regression [49]. Using an acute and chronic liver injury murine model, Ding *et al.* recently demonstrated that differential recruitment of proregenerative CXCR7-Id1 differs from that of profibrotic FGFR1-CXCR4 angiocrine pathways in LSECs to facilitate balance between liver regeneration and fibrosis [50].

#### 2.4.4. Angiogenesis

In the microenvironment of liver fibrosis with hepatic vasculature and tissue hypoxia alterations, HSCs produce multiple angiogenic factors, including vascular endothelial growth factor (VEGF), PDGF-BB, and angiopoietin 1 or 2, and express angiopoietin 1 receptors [51,52,53]. These angiogenic signals contribute to angiogenesis from neighboring endothelial cells by emitting paracrine signals and enhance HSC-induced fibrogenesis.

### 2.5. Reprogramming Metabolic Control to Regulate Hepatic Stellate Cells

Quiescent HSCs are similar to differentiated adipocytes in that they have abundant lipid droplets and they express lipogenic genes and transcriptional factors, which are downregulated upon HSC activation [54,55]. Recent data has shown that the transdifferentiation of quiescent HSCs into myofibroblasts induces glycolysis and causes lactate accumulation, which requires hedgehog signaling and the induction of HIF1-α for metabolic reprogramming of HSCs [56]. Hedgehog signaling controls the fate of the HSCs by regulating metabolism, engendering highly proliferative myofibroblastic phenotypes [56].

### 2.6. Autophagy, MicroRNA, and Epigenetic Regulation in Fibrogenesis

The activation of HSCs induces the loss of intracellular lipid droplets, the autophagic digestion of which may provide energy for cellular functions. A recent study demonstrated that hepatic fibrogenesis in mice requires the autophagy of activated HSCs [57]. Genetic and pharmacological inhibition of autophagy leads to the growth inhibition and downregulation of the fibrogenic properties of HSCs [57].

MicroRNAs (miRNAs) represent a family of small noncoding RNAs that control the translation and transcription of various genes. Recently, researchers have suggested that miRNAs modulate cellular processes in the liver, such as hepatocarcinogenesis. Several miRNAs are expressed in HSCs, controlling the fibrogenic process. The miR-29 family is significantly downregulated in mice with experimental fibrosis and in patients with advanced fibrosis [58]. In one study, this downregulation of miR-29 in cultured HSCs was mediated by TGF-β and the inflammatory signals LPS and NF-κB, and induced subsequent up-regulation of ECM genes [58]. *In vitro* experiments have indicated that the miR-19b mimic negatively regulates the TGF-β signaling components by reducing TGF-β receptor II and Smad3 expression, blocking TGF-β-induced expression of α1(I) and α2(I) procollagen mRNAs, and reducing α-SMA expression [59]. In addition, a study revealed that miR-19b expression is markedly downregulated in fibrotic rat and human livers [59]. MiR-221/222 expression is up-regulated in the human liver to a degree corresponding to the degree of liver fibrosis progression. Therefore, miR-221/222 may be a new marker for HSC activation and liver fibrosis progression [60].

Studies have indicated that the DNA methylation of genes expressed in quiescent HSCs contributes to maintenance of the quiescent phenotype [61]. A recently published study indicated that rat hepatic myofibroblasts induce heritable epigenetic changes in the sperm of the rats, thus attenuating fibrogenesis in their offspring. This result suggests that fibrogenic signals are epigenetically and genetically transmissible [62].

### 2.7. Microvesicles Containing Biomarkers for Fibrogenesis

Microvesicles (MVs) are 0.1- to 1.0-μm vesicles containing lipids, proteins, RNAs, and miRNAs that, which are formed by budding from the cellular plasma membrane. MVs have been implicated in many stages of liver disease, including liver fibrogenesis [63], and are possibly involved in fibrosis regression in the liver [64]. *In vitro* studies have indicated that the MVs released from T cells fuse with HSC membranes, leading to an up-regulation of *MMP* (*MMP1*, *MMP3*, *MMP9*, and *MMP13*) gene expression, down-regulation of the gene encoding procollagen-α1(I), and amelioration of TGF-β1 profibrogenic activity [64]. The CD147 molecule exposed on T-cell MVs contributes to HSC-induced fibrolytic activities [64]. A study suggested that the miRNAs contained in MVs promote fibrolysis [65]. Diehl *et al.* determined that cultured HSCs release MVs containing hedgehog ligands, which might promote fibrogenesis by increasing inducible nitric oxide synthase by primary sinusoidal endothelial cells *in vitro* [66].

### 2.8. Fibrosis Regression

Studies on rodent models have demonstrated that, once a liver injury is eliminated, fibrosis regression occurs. The restoration of fibrinolytic activities, such as the up-regulation of MMPs and downregulation of TIMPs, drives the reversal of fibrosis [67]. The clearance of activated HSCs or myofibroblast through apoptosis [67], senescence [48], or reversion into quiescent phenotypes is also crucial in fibrosis resolution [68,69].

Monocytes can promote resolution of fibrotic disease by (1) differentiating into regulatory macrophages that produce suppressor cytokines (e.g., IL-10); (2) producing MMPs that can degrade interstitial collagen directly; (3) locally depleting essential amino acids required for T-cell and myofibroblast proliferation; (4) actively promoting the apoptosis of myofibroblasts; and (5) phagocytosing ECM and cellular debris that would otherwise stimulate inflammatory and fibrogenic cell activation [3].

Several mechanisms have been implicated in the apoptosis of active HSCs: (1) the activation of death-receptor-mediated pathways (Fas or TNFR-1 receptors) and caspases 3 and 8; (2) the up-regulation of proapoptotic proteins (p53, Bax, caspase 9); and (3) the reduction of prosurvival genes (Bcl-2) [70].

During the course of fibrosis regression, the apoptosis of previously activated fibrogenic cells, which promote a favorable shift in the balance between fibrolytic MMPs and their profibrotic inhibitors within the microenvironment, leads to partial or even complete resolution of excess ECM accumulation [71]. In the recovery phase of liver fibrosis, macrophages harboring a distinct phenotype induce HSC apoptosis and produce active metalloproteinases to promote fibrosis resolution [72]. After prolonged injury, a liver with advanced fibrosis and cirrhosis exhibits more difficulty in undergoing tissue fibrinolysis. The reversibility of fibrosis potential tends to decline at advanced stages [16]. Although the limited resolution of advanced fibrosis is not well characterized, evidence has suggested that the fibrinolytic capability of MMPs decreases as the matrix stiffness and matrix cross-linking of collagen I in older septa increases [16]. This pathophysiological state may lead to a “point of no return” for livers with fibrosis [70].

## 3. Clinical and Molecular Aspects of Hepatitis-B- and Hepatitis-C-related Liver Fibrosis

### 3.1. Clinical Progression of Hepatitis-B- and Hepatitis-C-Related Liver Fibrosis

Several studies have reviewed the natural history of fibrosis progression. In two longitudinal observational studies in the late 1970s, of women who were infected by hepatitis C virus (HCV)-contaminated anti-D immune globulin, more than 95% of the patients exhibited mild to moderate hepatic inflammation according to liver biopsies 17 to 20 years postinfection. Approximately 55% of the patients were free of HCV infection, half exhibited evidence of fibrosis, and 2% to 4% had liver cirrhosis [73,74]. In another large cross-sectional study of 2235 HCV patients in Europe, the median rate of fibrosis progression was 0.133 fibrosis units (according to Metavir score) per year [75]. The host factors of age (older than 40), alcohol consumption (more than 50 g/day), and male sex were more strongly associated with fibrosis progression than the virological factors of HCV (e.g., genotype) [75]. The median estimated duration between HCV infection and the development of cirrhosis was 30 years, and this period was only 20 years in 33% of patients who underwent no treatment [75]. Patients with clinically significant hepatic fibrosis (Metavir stage ≥ 2 or Ishak stage ≥ 3) exhibited a higher risk of cirrhosis progression; therefore, antiviral therapy is required [76]. In addition, the stage of fibrosis predicts clinical outcomes, such as the incidence of liver transplantation and liver-related deaths [77]. However, published estimates of liver fibrosis progression in HCV infection vary. A recent metaanalysis of 111 studies reported the estimated prevalence of cirrhosis 20 years postinfection to be 16% and indicated that the duration of infection was the most consistent factor significantly associated with fibrosis progression [78].

Few studies have provided data on the natural course of fibrosis progression in hepatitis B. In a study of 57 patients in the immune-tolerant phase of the disease, the median rate of fibrosis progression was 0 fibrosis units/year in those with normal liver function and 0.21 fibrosis unit/year in patients with high alanine aminotransferase (ALT) levels (progression into the immune clearance phase) [79]. In another study of 128 HBeAg-positive patients with paired liver biopsies, spontaneous sustained disease remission (HBeAg seroconversion and hepatitis B virus (HBV)-DNA < 10,000 copies/mL) and a younger age (20–29 years) were associated with minor fibrosis progression and even regression [80].

### 3.2. Factors Contributing to Hepatitis-B-Related Fibrosis and Cirrhosis

Two factors contribute to liver cirrhosis induced by HBV: an HBV viral factor and HBV-induced hepatic inflammation (Table 2). The first viral factor is HBV genotype; HBV genotype C is associated with disease progression to cirrhosis or hepatocellular carcinoma (HCC) and poorer responses to interferon-based treatments compared with genotype B [81,82]. The second viral factor is the HBV viral load or HBV-DNA level. The REVEAL-HBV study in Taiwan enrolled 3582 untreated HBV carriers without cirrhosis and conducted followed-up observation for a mean of 11 years. Among 365 patients newly diagnosed with cirrhosis, the cumulative incidence of cirrhosis in those with an HBV-DNA level lower than 300 copies/mL was 4.5%, whereas that in patients with an HBV-DNA level greater than 10^6^ copies/mL (*p* < 0.001) was 36.2% [83]. After the researchers adjusted the HBeAg status and serum ALT levels, the HBV-DNA level was the strongest predictor of progression to cirrhosis; specifically the relative risks associated with HBV-DNA levels ≥10^4^–10^5^, 10^5^–10^6^, and ≥10^6^ copies/mL were 2.5, 5.6, and 6.5, respectively [83]. According to this landmark study, a high baseline serum HBV-DNA level predicts a high risk of cirrhosis and HCC development [83,84]. Using a newly developed pyrosequencing technique to quantify basal core promoter mutation [85], researchers recently recognized that a higher proportion of BCP mutants are associated with an increased risk of liver cirrhosis development in HBV carriers with genotype B or C infection [86], suggesting that the fibrogenesis of chronic hepatitis B possibly involves viral mutation.

**Table 2:** Vial factors associated with viral hepatitis related fibrosis and cirrhosis.

Viral Factors	Hepatitis B	Hepatitis C
Viral genotypes	Genotype C associated with cirrhosis compared with genotype B	Genotype 1 associated with cirrhosis
Viral load	Higher viral load associated with fibrogenesis and HCC	Higher viral load associated with HCC
Viral mutation	Basal core mutation	Unknown
Viral proteins	HBx protein	Unknown

In addition, the HBV viral protein correlates with fibrosis progression. Martin-Vilchez *et al.* discovered that the expression of the HBx protein in hepatocytes results in paracrine activation and the proliferation of HSCs. In both humans and rats, primary HSCs exposed to a conditioned medium derived from HBx-expressing hepatocytes increased the expression of type 1 collagen, CTGF, α-SMA, MMP-2, TGF-β, and enhanced the cell proliferation rate [87]. This HBx-induced HSC activation could be prevented by neutralizing the TGF-β antibody, indicating the involvement of TGF-β in the fibrogenetic process [87]. Similarly, Guo *et al.* confirmed that the HBx protein induced the proliferation of LX-2 cells (a human HSC) by using a coculture system. A cell cycle study reported that HBx accelerated the progression of G1 to S in LX-2 cells as the expression of α-SMA, TGF-β1, TGF-β RII, CTGF, and type 1 collagen increased. These results suggest that HBx facilitates liver fibrosis by promoting HSC proliferation and up-regulating the expression of fibrosis-related molecules [88]. By contrast, another *in vitro* study reported that low concentrations of HBV (1.0 × 10^3^–6.2 × 10^4^ copies/mL) or HBs proteins (0.04–0.62 μg/mL) promoted the proliferation of LX-2 cells, whereas high concentrations of HBV (1.2 × 10^5^–5.0 × 10^5^ copies/mL) or HBs proteins (1.25–20 μg/mL) inhibited the proliferation of LX-2. HBV can transiently infect and replicate in cultured LX-2 cells and express HBs and HBc *in vitro*. HBV infection significantly increases the mRNA and protein expression of type 1 collagen in LX-2 cells [89].

Jin *et al.* developed a CCl_4_-induced liver fibrosis model in HBV-transgenic (HBV-Tg) mice. Liver fibrosis spontaneously developed in the HBV-Tg mice with an elevated transcription of type 1 collagen, MMP-2, and TIMP-1. After chronic CCl_4_ treatment, the HBV-Tg mice exhibited an increase in serum ALT, liver fibrosis, and cirrhosis levels compared with the wild mice controls. Moreover, the transcription of type 1 collagen and MMP2, level of TIMP-1, and activation of HSCs increased significantly in the livers of the CCl_4_-treated HBV-Tg mice. In addition, the number of hepatic NKT cells increased after CCl_4_ treatment, and NKT cells were overactivated in HBV-Tg mice in the long term. The inflammatory cytokines IL-4 and IL-13 produced by NKT cells played a pivotal role in HSC activation in an *in vitro* coculture experiment. These data suggested that NKT cells from HBV-Tg mice induce HSC activation in liver fibrogenesis [90]. Another recent study demonstrated that IL-22-pathway-associated genes are significantly up-regulated in HBV-infected liver tissues, and IL-22^+^ cells in the liver positively correlate with liver fibrosis severity [91]. In an HBV-Tg mouse model of T-cell-mediated chronic liver inflammation and fibrosis, the blockade of IL-22 attenuated the hepatic expression of CXCL10 and CCL20 and subsequently reduced Th17 cell recruitment and liver fibrogenesis [91]. The IL-22 treatment stimulated HSCs to secrete chemokines, promoting Th17 chemotaxis *in vitro*. Thus, IL-22 played a crucial role in exacerbating chronic liver inflammation and fibrosis by recruiting hepatic Th17 cells in HBV-infected patients and HBV-Tg mice [91].

In addition to the HBV viral factors, hepatic inflammation is another key factor in fibrogenesis. Chronic inflammation leads to fibrosis and cirrhosis [92], both of which are less likely to occur if no inflammation is present. For example, in the immune-tolerant phase of chronic hepatitis B, patients exhibit high HBV viremia titers; however, in the absence of active inflammation, these patients do not have liver fibrosis [80].

### 3.3. Viral Factors for Hepatitis-C-Related Fibrosis and Cirrhosis

In chronic hepatitis C, the roles of HCV genotypes and HCV RNA levels in predicting disease progression are less clear than those in chronic hepatitis B (Table 2) [75]. Two previous studies have demonstrated that cirrhosis progression and the development of HCC is associated with the HCV genotype 1b [93,94]. Another study determined that the HCV RNA level predicts end-stage-liver-disease-related death among IV drug users with chronic HCV infection [95]. A recent community-based prospective study followed 925 anti-HCV positive participants during 1991 and 2006. Fifty-five participants developed HCC over 8476 person-years of follow-up, representing an incidence rate of 648.9 per 100,000 person-years. The cumulative HCC risk was 1.1% in those with an HCV RNA seronegative status, whereas it was 6.4% in those with low HCV RNA levels and 14.7% in those with high HCV RNA levels (*p* < 0.001). In addition, the cumulative risk increased as the serum ALT levels increased; specifically, the risk for patients of whom the serum ALT levels were persistently ≤15 U/L was 1.7%, whereas that for patients of whom the ALT level was ever ≥45 U/L was 13.8% (*p* < 0.001). HCV genotype 1 was more strongly associated with a higher cumulative HCC risk (12.6%) than other HCV genotypes (4.5%; *p* < 0.001). Elevated serum levels of HCV RNA and ALT, and HCV genotype 1 infection were independent risk predictors of HCC [96]. However, this study did not investigate the risk predictors for liver cirrhosis.

A recent study investigated TGF-β1 and IL-13 signaling in various chronic liver diseases and determined that phospho-Smad3 staining correlated significantly with the fibrotic stage in patients with chronic HBV infection and steatohepatitis, whereas IL-13 positive immunostaining correlated with the fibrotic stage in those with HCV infection and steatosis/steatohepatitis. Compared with healthy controls, HCV patients exhibited significantly elevated levels of the hepatic IL-13 protein and serum IL-13. Therefore, depending on the cause of liver damage, TGF-β or IL-13 signaling are predominant factors of HBV- and HCV-related liver fibrogenesis, respectively [97].

### 3.4. Host Factors Associated with Fibrosis Progression to Hepatocellular Carcinoma

In addition to viral factors, several host factors are associated with liver cirrhosis and HCC progression. Several genetic studies have identified that a high-risk single nucleotide polymorphism predicts fibrosis progression. A cirrhosis risk score (CRS) signature consisting of seven markers more accurately differentiates high- and low-risk cirrhosis in Caucasian CHC patients than clinical factors do [98]. In addition, this CRS facilitates predicting fibrosis progression in men with initially mild chronic HCV [99].

Risk factors that accelerate the clinical progression of HCV include alcohol intake, coinfection with HIV or HBV, the male sex, and an older age at infection. Once cirrhosis is established, the risk of HCC increases approximately 1% to 4% per year [100,101]. A recent community-based cohort study indicated that extreme obesity (BMI ≥ 30) was independently associated with a fourfold risk of HCC (RR: 4.13; 95% CI: 1.38–12.4) in HCV carriers but not in HBV carriers (RR: 1.36; 95% CI: 0.64–2.89). Diabetes was associated with an increased risk of HCC in both HCV carriers (RR: 3.52; 95% CI: 1.29–9.24) and HBV carriers (RR: 2.27; 95% CI: 1.10–4.66). HCC risk increased more than 100-fold in HCV carriers who were both obese and had diabetes [102]. In a long-term prospective study on 352 patients with compensated HCV-induced cirrhosis, Bruno *et al.* determined that the presence of esophageal varices at the baseline predicts decompensation and mortality. The development of HCC during follow up greatly hastens decompensation, which is the main determinant of death. Patients with a MELD score ≤10 upon joining the study had a prolonged life expectancy [103].

## 4. Treatment for Viral Hepatitis-Related Liver Fibrosis and Cirrhosis

Several therapeutic strategies can be applied to treat hepatic fibrosis: eliminating the causes of injury and their mediators; reducing inflammation and the immune response; targeting specific signaling: receptor–ligand interaction and intracellular signaling; inhibiting matrix synthesis and increasing scar matrix degradation; and stimulating HSC apoptosis and providing bone marrow or cell transplantation [3]. However, eliminating the underlying causes of liver diseases is the most crucial and effective antifibrotic therapy (Table 1).

### 4.1. Antifibrotic Effect of Antiviral Therapy

Because viral infection is critical in the progression of liver disease, antiviral therapy is crucial to successfully preventing diseases of the liver. Cirrhosis can even regress as long as the etiologic agent can be cleared (HCV) or the disease process can be controlled (HBV) (Table 3).

**Table 3:** Beneficial results of successful antiviral therapy in viral hepatitis.

Outcomes	Hepatitis B	Hepatitis C
Fibrosis regression	Yes	Yes
Reduction of liver disease progression	Yes	Yes
HCC reduction	Yes	Yes
Reduction of liver related mortality	Probable	Probable

Five oral nucleos(t)ide analogs (lamivudine, adefovir dipivoxil, entecavir, telbivudine, and tenofovir disoproxil fumarate) and two immunomodulators (conventional and pegylated interferons) can facilitate HBV treatment. Short-term treatment goals include ALT normalization (biochemical response), HBeAg seroconversion, HBV DNA negativity (virological response), and improvement of hepatitis activity or fibrosis (histological response). Medium-term treatment goal are HBsAg loss and seroconversion, and the long-term goal is liver cirrhosis, HCC, and liver-related death minimization. The current criteria for initiating anti-HBV therapy include having active hepatitis (abnormal ALT levels) and compensated or decompensated cirrhosis with HBV viremia [104].

Growing evidence has indicated that prolonged oral anti-HBV therapy may stop liver inflammation, reduce fibrogenesis, and even reverse fibrosis. Paired liver biopsy before and after therapy of 57 patients who received entecavir for more than three years indicated that 96% of the patients histologically improved (a greater than 2-point decrease in the Knodell necroinflammatory score and no increase in the Knodell fibrosis score), and 88% of the patients, including all 10 patients with baseline advanced fibrosis or cirrhosis, exhibited fibrosis improvement (a greater than 1-point improvement in the Ishak fibrosis score) [105]. Among 348 patients receiving long-term tenofovir therapy, 87% exhibited histological improvement and 51% exhibited fibrosis regression. Of the 96 patients with baseline cirrhosis, 74% no longer exhibited cirrhosis after 240 weeks of therapy [106].

Interferon-based therapy was the mainstay of HCV therapy before the era of direct antiviral agents. In one study, interferon-α therapy exhibited antifibrotic activity by inhibiting the production of TGF-β, reducing HSC activation, and stimulating HSC apoptosis *in vitro* [107]. Another study observed the antifibrotic effects that interferon-γ exerted in liver cells through STAT-1 phosphorylation, the up-regulation of Smad7, and impaired TGF-β signalling [108]. In a clinical study on 593 chronic HCV patients receiving a two- to six-month course on interferon monotherapy in Japan, a sustained response to therapy was associated with a mean reduction in the fibrosis score of 0.88 after three years or more of follow up. The rate of fibrosis regression was 0.28 fibrosis unit/year [109]. A French study enrolled 96 biopsy-proven cirrhotic patients receiving interferon-based treatment; fibrosis regression was observed in 18 patients by conducted paired biopsies and associated with decreased liver-related morbidity and mortality [110]. After the researchers introduced highly effective direct antiviral agents, they anticipated long-term antifibrotic effects.

These effective antiviral agents for hepatitis B and hepatitis C can persistently suppress viral replication and even cure the disease [111,112]. Early identification of patients at risk of cirrhosis and prompt antiviral treatment is vital to preventing disease progression to liver cirrhosis.

### 4.2. Antiviral Therapy Halts Disease Progression in Cirrhotic Patients

A clinical trial by Liaw *et al.* provided the first evidence that anti-HBV therapy slows the progression of advanced liver disease. Continual treatment with lamivudine delayed the clinical progression of chronic HBV, advanced fibrosis, and cirrhosis by significantly reducing the incidence of hepatic decompensation and the risk of HCC [113]. In a metaanalysis of 21 studies in which chronic hepatitis B patients were exposed to nucleos(t)ide analogues for more than 24 months, HCC was diagnosed in 2.8% and 6.4% of the treated and untreated patients, respectively, after a 46 (32–108)-month period (*p* = 0.003), in 10.8% and 0.5% of nucleos(t)ide naive patients with and without cirrhosis, (*p* < 0.001), and in 17.6% and 0% of lamivudine resistance patients with and without cirrhosis (*p* < 0.001) [114]. HCC developed less frequently in treatment-naive patients than in those without virological remission (2.3% *vs.* 7.5%, *p* < 0.001) [114]. The chronic hepatitis B patients receiving medium-term nucleos(t)ide analog therapy had a significantly lower incidence of HCC compared with the untreated patients; nevertheless, treatment did not completely eliminate the HCC risk [114].

In a recently published large-scale study conducted in Japan, 472 patients treated with entecavir and 1143 treatment-naïve controls were enrolled. After matched propensity score analysis, the cumulative HCC incidence rates at 5 years were 3.7% and 13.7% for the entecavir and control groups, respectively (*p* < 0.001). After adjustment for known HCC risk factors, the entecavir treatment group was less likely to develop HCC than the control group (HR: 0.37, 95% CI: 0.15–0.91, *p* = 0.030). The greatest HCC risk reduction was observed in high-risk patients, especially those with cirrhosis. Furthermore, the HCC suppression effect was greater in entecavir-treated patients (*p* < 0.001) than in lamivudine-treated cirrhosis patients (*p* = 0.019) with drug resistance when they were compared with the control groups [115]. This study suggested that antiviral therapy reduces the development of HCC only in patients at a higher risk of HCC (cirrhotic patients, older patients, or patients with active hepatitis), and not in noncirrhotic patients, probably because of the suboptimal follow-up period. The major effect of the therapy was persistent viral suppression. Another study in Hong-Kong examined 1446 chronic hepatitis B patients who had been treated with entecavir for more than 12 months and 424 treatment-naïve patients. The primary endpoint was the five-year cumulative probability of hepatic events, defined as any cirrhotic complication, HCC, and liver-related mortality. The two groups did not exhibit significant difference in hepatic events. However, among patients with liver cirrhosis (482 entecavir treated and 69 treatment naïve), the treated patients, particularly those who had maintained viral suppression, exhibited a lower risk of hepatic events (HR: 0.51, 95% CI: 0.34–0.78), HCC (HR: 0.55, 95% CI: 0.31–0.99), liver-related mortality (HR: 0.26, 95% CI: 0.13–0.55), and all-cause mortality (HR: 0.34, 95% CI: 0.18–0.62) than did the untreated patients [116]. Through a multicenter European study investigating 372 entecavir-treated patients (26% with cirrhosis) with a median follow up of 20 months, Zoutendijk *et al.* revealed that virological responses (HBV-DNA < 80 IU/mL) were associated with a lower probability of disease progression, especially in cirrhotic patients (HR: 0.22, 95% CI: 0.05–0.99; excluding decompensated patients). The protective effect of entecavir was reduced when incomplete viral suppression exceeded a threshold of 2000 IU/mL [117].

Poynard *et al.* pooled 3010 naïve patients having pre- and posttreatment liver biopsies from four clinical trials, and observed that therapy with a combination of pegylated interferon and ribavirin significantly reduced the fibrosis progression rate of patients with chronic hepatitis C, and cirrhosis reversal was observed in 49% (75/153) of the patients with baseline cirrhosis [118]. A study in Taiwan examined 116 biopsy-proven cirrhotic patients receiving interferon α-2b and ribavirin treatment over a median follow-up period of 37 months. Eleven patients without sustained virologic response (SVR) and five patients with SVR (*p* = 0.018) developed HCC, indicating that achieving SVR may reduce HCC incidence [119]. In a retrospective study conducted in Italy that examined 920 HCV-related cirrhosis patients, SVR reduced liver-related complications and mortality after interferon monotherapy and lowered (but did not eliminate) the HCC risk [120]. A prospective 12-year follow-up study of 218 compensated HCV-induced cirrhotic patients in Italy indicated that SVR achievement after interferon therapy prevented the development of esophageal varices [121]. A retrospective study that examined 568 HCV patients with liver cirrhosis undergoing pegylated interferon-α and ribavirin treatment indicated that SVR was achieved in approximately one-third of patients with HCV-related cirrhosis, independently reducing the likelihood of clinical decompensation and improving survival [122]. SVR in HCV patients markedly reduces clinical complications and improves portal hypertension [123]. However, after SVR, patients should be monitored because some remain at risk of liver disease complications, particularly HCC [124]. Another study examined 130 biopsy-proven cirrhosis patients; after patients underwent interferon-based therapy over a mean follow up of 6.4 years, multivariate analysis revealed that SVR was associated with a lower HCC risk (HR: 0.09; 95% CI: 0.01–0.77), liver transplantation (HR: 0.04; 95% CI: 0.003–0.63), and improved survival [125]. A recent metaanalysis of 18 observational studies indicated that, in HCV carriers, SVR was associated with a reduced HCC risk (relative risk: 0.24, 95% CI: 0.18–0.31) [126].

### 4.3. Direct Antifibrotic Therapy Using Nonantiviral Agents

#### 4.3.1. Conventional Medications

Statins, angiotensin-converting enzyme inhibitors (ACEI), angiotensin-II receptor blockers (ARB), and pioglitazone have antifibrotic properties. A previous study indicated that early atorvastatin treatment inhibited HSC activation and fibrosis in a rat model of bile-duct-ligation-induced fibrosis, whereas late treatment reduced HSC turnover and activity [127]. Recently, several studies have focused on the association between the renin-angiotensin system and liver fibrosis, indicating that ACEI and ARB exhibit potential antifibrotic effects [128]. In addition, one study indicated that pioglitazone improves hepatic fibrosis by activating the AMP-activated protein kinase pathway in rats with nonalcoholic steatohepatitis (NASH) [129].

#### 4.3.2. Molecular Target Agents: The Example of Sorafenib

Sorafenib is a small-molecule inhibitor of tumor-cell proliferation and angiogenesis that induces apoptosis in various tumor cells [130,131]. It inhibits the serine-threonine kinases Raf-1 and B-Raf as well as the receptor tyrosine kinase activity of VEGFRs 1, 2, and 3, and PDGFR-β [130,131]. The Raf-1 and VEGF pathways have been implicated in the molecular pathogenesis of HCC [132,133]. Two large phase-3 clinical trials have recently reported improvements in median survival and time to radiologic progression in advanced HCC patients treated with sorafenib [134,135]. Currently, sorafenib is the only FDA-approved agent for advanced stage HCC [136]. Inhibition of the PDGF receptor pathway, was recently demonstrated the potential antifibrotic effect of sorafenib both *in vitro* and in a rat model of experimental fibrosis. Sorafenib reduced proliferation, induced apoptosis, increased the ratio of MMPs to TIMPs, and reduced collagen synthesis in HSCs. Furthermore, sorafenib inhibited the phosphorylation of ERK, Akt, and p70S6K both *in vitro* and *in vivo* [137]. Hong *et al.* confirmed the antifibrotic effect of sorafenib at lower doses *in vivo* and *in vitro*; however, they observed that sorafenib was ineffective in established cirrhosis. Therefore, the dosage of antifibrotic sorafenib for humans should be lower than that used in HCC therapy to improve tolerability and compliance [138]. Future clinical studies should explore the antifibrotic efficacy of sorafenib in patients.

#### 4.3.3. Ongoing Clinical Trials of Molecular Target Agents

Several humanized antibodies against the TGF-β family have been evaluated, including fresolimumab (GC1008, Genzyme), which is undergoing clinical trials for the treatment of idiopathic pulmonary fibrosis (IPF), focal segmental glomerulosclerosis, and myelofibrosis. STX-100 (Stromedix) is a humanized anti-α_v_β_6_ integrin antibody, inhibiting the latent precursor form of TGF-β activation and currently undergoing phase II clinical trials for treating IPF. The lysyl oxidase 2 (LoxL2) belongs to a family of matrix enzymes responsible for collagen cross-linking. The blocking of LoxL2 activity in murine models reduces myofibroblast levels, ECM production, and TGF-β expression [139]. Simtuzumab (GS-6624, Gilead) is a noncompetitive allosteric antibody inhibitor of LoxL2 undergoing clinical development for the treatment of fibrotic diseases and cancer, and is undergoing phase II studies of NASH-associated advanced fibrosis and cirrhosis patients as well as primary sclerosing cholangitis patients.

Pirfenidone (InterMune) inhibits the proliferation of HSC induced by PDGF in rats through the inhibition of PKC-mediated Na^+^/H^+^ exchange activation and significantly suppresses TGF-β-induced type I collagen production [140]. The phase III CAPACITY clinical trials concluded with a favorable benefit-risk profile, indicating that pirfenidone is an appropriate treatment option for patients with IPF [141]. It has been approved in Europe and Japan, and the phase III ACSCEND trial on IPF has recently been completed, yielding positive results [142].

Several anti-IL-13 antibodies, including tralokinumab (MedImmune) and SAR156597 (Sanofi; targeting both IL-13 and IL-4), are undergoing investigation in IPF studies. PRM-151 (Promediator) is a human recombinant form of pentraxin-2 (serum amyloid P) that regulates innate immune responses and inhibits the differentiation of monocytes into M2 macrophages [143]; it is currently the subject of a phase II study for myelofibrosis.

Nintedanib (BIBF 1120, Boehringer Ingelheim) is a multitarget tyrosine kinase inhibitor that inhibits platelet derived growth factor receptor (PDGFR), fibroblast growth factor receptor (FGFR), and vascular endothelial growth factor receptor (VEGFR). Nintedanib showed promise in treating IPF patients in a phase II and III trial [144,145]. Preclincal research suggested that blocking connective tissue growth factor (CTGF) activity with siRNA reduces liver fibrosis and preserves liver function [146]. FG-3019 (FibroGen), a humanized antibody against CTGF, has been tested for treating chronic-hepatitis-B-related liver fibrosis (ClinicalTrials.gov NCT01217632).

### 4.4. Tissue-Specific Targeting

Targeting the core pathway in hepatic fibrogenesis is possible; however, researchers have been cautious because of the potential off-target effects in nonfibrotic or unaffected tissues. For example, targeting the TGF-β pathway would inhibit fibrogenesis, but could also cause chronic inflammation and even impair tumor suppression [3]. Drugs can be designed to specifically target HSCs in higher doses to prevent toxicity from spreading to other cells. As HSCs take up vitamin A with a retinol-bound protein, Sato *et al.* showed that vitamin-A-coupled liposomes against gp46 successfully ameliorated collagen deposition in experimental liver fibrosis models [147]. During liver fibrogenesis, mannose 6-phosphate (M6P)/insulin growth factor type II receptor was *de novo* expressed in activated HSCs. Moreno *et al.* incorporated losartan into M6P-modified human serum albumin for an antifibrotic treatment in an experimental liver fibrosis model and observed that this HSC-targeted losartan markedly reduced advanced liver fibrosis [148].

## 5. Perspectives

Various causes of hepatic fibrogenesis have been identified, including viral infection, alcohol abuse, steatohepatitis, metabolic disorders, and immune attack. The simplest approach to targeting liver fibrosis is disease prevention. For example, immunization can prevent hepatitis B, transmission precautions can be taken for hepatitis C, abstinence from alcohol can prevent alcoholic hepatitis, and a healthy diet, lifestyle modification and body weight reduction can prevent steatohepatitis. The next step in combatting liver diseases is to eliminate the underlying causes through medical control methods, such as antiviral therapy, which can control HBV or even eradicate HCV. Recently, the FLINT trial reported that NASH was successfully treated using obeticholic acid, reducing the NAFLD Activity Score by at least 2 points and yielding no exacerbation of fibrosis (ClinicalTrials.gov NCT01265498).

For established cirrhosis or diseases without a standardized therapy (e.g., alcoholic cirrhosis and cryptogenic cirrhosis), antifibrotic therapies targeting the central pathway of fibrogenesis are the only potential treatment. Although numerous studies have investigated the pathobiology of fibrogenesis and identified potential therapeutic targets, there is still a large gap between the bench and the bedside, and few clinical trials have explored positive preclinical signals. Several major obstacles to developing antifibrotic therapies include slow fibrogenesis progression, lacking validated biomarkers, and insensitive clinical endpoints [3]. Nevertheless, antifibrotic therapy has recently become the focus of investigation and remains a critical unmet clinical need. Several new agents have been proved to exhibit antifibrotic efficacy in IPF [141,142,143,144,145]. In addition to antiviral therapies for controlling viral hepatitis, future clinical trials are required to investigate direct antifibrotic therapies that can halt fibrogenesis progression, HCC development, and death.

## 6. Conclusions

Viral hepatitis B and hepatitis C are major health problems worldwide that result in critical liver fibrosis or cirrhosis after long-term infection. Several viral and host factors have been identified to predict the disease progression. Growing evidence has suggested that current antiviral therapies can effectively control or even eradicate HBV and HCV, and subsequently ameliorate or even reverse cirrhosis, cirrhotic complications, and HCC. However, more effort should be exerted in developing direct antifibrotic agents for patients with established cirrhosis who cannot benefit from antiviral therapies.
